# Reconstruction of Periodic Band Limited Signals from Non-Uniform Samples with Sub-Nyquist Sampling Rate

**DOI:** 10.3390/s20216246

**Published:** 2020-11-02

**Authors:** Dongxiao Wang, Xiaoqin Liu, Xing Wu, Zhihai Wang

**Affiliations:** School of Key Laboratory of Vibration and Noise under Ministry of Education of Yunnan Province, Kunming University of Science and Technology, Kunming 650500, China; dxwang@stu.kust.edu.cn (D.W.); xwu@kust.edu.cn (X.W.); wzh@kust.edu.cn (Z.W.)

**Keywords:** servo data, signal reconstruction, nonuniform sampling, equivalent sampling

## Abstract

Important state parameters, such as torque and angle obtained from the servo control and drive system, can reflect the operating condition of the equipment. However, there are two problems with the information obtained through the network from the control and drive system: the low sampling rate, which does not meet the sampling theorem and the nonuniformity of the sampling points. By combing equivalent sampling and nonuniform signal reconstruction theory, this paper proposes a reconstruction method for signal obtained from servo system in periodic reciprocating motion. Equivalent sampling combines the low rate and nonuniform samples from multiple periods into one single period, so that the equivalent sampling rate is far increased. Then, the nonuniform samples with high density are further resampled to meet the reconstruction conditions. This step can avoid the amplitude error in the reconstructed signal and increase the possibility of successful reconstruction. Finally, the reconstruction formula derived from basis theory is applied to recover the signal. This method has been successfully verified by the simulation signal of the robot swing process and the actual current signal collected on the robot arm testbed.

## 1. Introduction

As a commonly used method of condition monitoring and fault diagnosis, vibration monitoring is very common and effective. This method requires the installation of the vibration sensor for data collection, which is sometimes difficult to implement and costly. In addition, the location of the sensor affects its sensitivity to signals [1]. Due to the deficiency of vibration monitoring, non-invasive diagnostic methods such as motor current signature analysis (MCSA) [2] and load torque signature analysis (LTSA) [3] have been further developed. For recent servo drive systems, this kind of important information such as current, angle, etc. can be obtained directly from the servo control drive system through the certain communication method such as EtherCAT, CAN, etc. Ehter CAT and CAN (Controller Area Network) are two efficient bus communication technologies.Typical cases include ePS [4], a state monitoring and remote diagnosis platform for machine tools used by Siemens, ABB’s Remote Service platform [5], and FANUC’s remote monitoring system for industrial robots [6]. These platforms can all collect the servo data during the operation of the equipment through the certain interfaces. Although the torque signal cannot be read directly, it can be identified by the electrical parameters of the drive system [7]. Therefore, it is very important to analyze the servo data obtained from the network. However, the signal obtained through the network is different from isochronous sampling. The data density is low, and the sampling interval is uneven. The information obtained is limited because of the two characteristics. In response to these problems, the equivalent sampling theory which can improve the sampling rate and the reconstruction theory of nonuniform sample points are focused on in this paper.

The development of equivalent sampling technology breaks through the limitation of Nyquist sampling theorem. It can sample the signal at a rate much lower than the Nyquist sampling rate while retaining the information of the original signal [8,9]. As a basic method of equivalent sampling technology, random equivalent sampling (RES) is widely used in signal sampling because of its low cost and high accuracy [10,11]. The basic idea of RES is to reconstruct the multi-cycle samples in a fast, equivalent period. However, due to the randomness of the time interval between the trigger position and the sampling clock, the sample distribution is not uniform. In order to obtain a sufficiently high reconstruction accuracy, the RES must undergo a large amount of random sampling to reconstruct the original signal [12]. This multiple sampling will generate a large number of redundant samples which will greatly reduce the reconstruction efficiency. In this paper, by extracting the period of the signal to be reconstructed, the principle of equivalent sampling is used to recombine multi-period nonuniform samples within one period to improve the sampling rate. However, the distribution of the reorganized sample points is still uneven. To solve this problem, we need to utilize the theory of nonuniform reconstruction.

Early research on nonuniformly sampled signals mainly focused on the possibility of reconstruction and theoretical derivation of reconstruction conditions. Black [13] first summarized the reconstruction conditions of non-uniform samples on the basis of the research of Cauchy [14]. Since the statement is not precise enough, Yen [15] further perfected the theory of non-uniform sampling. He pointed out that when a band-limited time continuous signal with the highest frequency component of W is divided into several equal intervals with a width of N/(2 W), and N instantaneous sample points are taken from each interval in any manner, the signal can be uniquely determined. In the underspecified situation of sampling points less than N and the over-specified situation of sampling points greater than N, certain conditions must be met before the signal can be determined. At the same time, he also discussed the situation of recurrent nonuniform sampling [16,17], which is a typical situation in digital signal processing. So, as to realize the transition of the reconstruction algorithm from theory to engineering application, Ouderaa [18] proposed a reconstruction formula for non-uniform sampling of finite points. The construction of filter banks for the reconstruction of nonuniform band-limited signals has also been widely developed [19,20]. Wiley et al. [21] applied the iterative method to the field of nonuniform sampling for the first time. The proof that the iterative method can be used to recover the original band limited signal from nonuniform samples is given by Marvasti in [22]. Iterative methods often require a large number of iterations to achieve high reconstruction accuracy. Based on the theory of bases and frames, E. Margolis proposes a noniterative reconstruction formula suitable for any number of points and discusses the stability of the reconstruction algorithm [23].

Equivalent sampling solves the problem of signal sampling and reconstruction at the sub-Nyquist sampling rate, but this theory has requirements for the sampling process. This paper uses the method of period extraction to realize the recombination of sampling points in one period, which can effectively increase the sampling rate and is not restricted by the sampling process. In addition, this paper proposes a down-sampling rule to screen the reorganized samples, which improves the possibility of successful reconstruction. By researching the reconstruction of non-uniformly distributed sample points with sub-Nyquist rate, this paper provides a method for the reconstruction of the signal obtained by the servo system through the certain network interfaces.

## 2. Basic Theories

### 2.1. Equivalent Sampling

Equivalent sampling means that for a signal with a fixed period, under the condition of a low sampling rate, the purpose of increasing the sampling rate can be achieved by combining multi-period signals. According to the main characteristics, equivalent sampling can be divided into two forms: sequential equivalent sampling (SES) and random equivalent sampling.

In SES process [8], each period of a signal with a period of $T_{a}$ is equally divided into n parts. Obviously, the time interval $\Delta t=T_{a}/n$. At time $kT_{a}+\left(m-1\right)\Delta t$, the *m*th sampling is performed by shifting the sampling time of each cycle backward by Δt. In the above sampling process, due to the sampling interval $\Delta t\ll T_{a}$, it does not satisfy the sampling theorem. But after *m*th of sampling, we can get a complete period waveform by rearranging the sampling points in one cycle.

**Figure 1:**
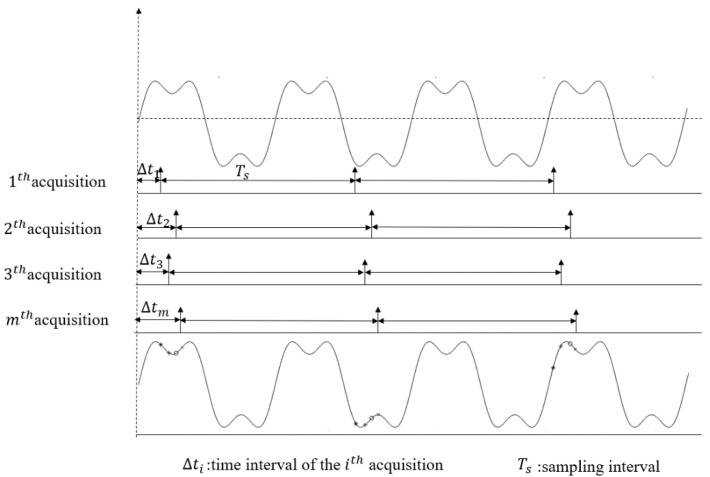
Schematic Diagram of random equivalent sampling (RES).

The principle of RES [12] is shown in Figure 1, the sampling circuit performs one sampling under the control of the sampling clock from the position of the random time interval Δt of the trigger level. The time interval ∆*t* between the first sampling clock and the trigger position of each sampling is distributed on [0 $T_{s}$] and is random, where *T_s_* is the period of the sampling clock. Then, according to this random but definite time interval ∆*t*, the position of each sample point is determined in the recombined signal. 

For periodic signal, the equivalent sampling technology is not limited by Nyquist sampling theorem. It reduces the sampling rate at the cost of the increase of sampling time by reorganizing the multiple sampling data. 

### 2.2. Reconstruction of Nonuniform Samples

The theory of frames and bases is a kind of framework for the study of nonuniform sampling. E. Margolis et al. [23] proposed an effective reconstruction algorithm based on this theory. This algorithm is briefly introduced in two aspects: the theory of frames and bases and the reconstruction method.

#### 2.2.1. Frames and Bases

Let *x*(*t*) be the periodic band limited signal to be recovered, and ${\left\{x\left(t_{i}\right)\right\}}_{i=1}^{N}$ be its N non-uniform samples. The basic idea of the reconstruction algorithm is to represent x(t) as a linear combination of functions $\varphi_{i}\left(t\right)$, i.e.,

(1) $$x\left(t\right)=\overset{N}{\underset{i=1}{\sum }}x\left(t_{i}\right)\varphi_{i}\left(t\right)$$

The functional space $V_{\varphi }$ is defined as

(2) $$V_{\varphi }=\left\{\sum_{i=1}^{N}c_{i}\varphi_{i}\left(t\right)|c\in R^{N}\right\}$$

If there are constants A > 0 and B < ∞ that can make all $x\left(t\right)\in V_{\varphi }$ satisfy Equation (3), then the set of functions ${\left\{\varphi_{i}\left(t\right)\right\}}_{i=1}^{N}$ is described as a frame for $V_{\varphi }$.

(3) $$A{\left\|x\left(t\right)\right\|}^{2}\leq \overset{N}{\underset{i=1}{\sum }}{\left|x\left(t\right),\varphi_{i}\left(t\right)\right|}^{2}\leq B{\left\|x(t)\right\|}^{2}$$

Here,a(t),b(t) is an inner product of two functions a(t) and b(t). ${\left\|a\left(t\right)\right\|}^{2}$ is the squared norm of a(t). The constants A and B are called the frame bounds, and they can be determined by the eigenvalues of the correlation matrix R (Equation (4)). A is the minimum value of all eigenvalues, and B is the maximum one.

(4) $$R_{ij}=\langle \varphi_{i}\left(t\right),\varphi_{j}\left(t\right)\rangle$$

In special cases, if N=M, ${\left\{\varphi_{i}\left(t\right)\right\}}_{i=1}^{N}$ are linearly independent of each other, they constitute a basis of space $V_{\varphi }$. 

#### 2.2.2. Reconstruction Method

Let x(t) be a signal with period T and band limited to 2πK/T. Then, x(t) can be reconstructed by its N≥2K+1 unevenly distributed samples as

(5) $$x\left(t\right)=\overset{N-1}{\underset{p=0}{\sum }}x\left(t_{p}\right)h_{p}\left(t\right)$$

In this reconstruction formula, when N is an even number,

(6) $$h_{p}\left(t\right)=\cos\left(\frac{\pi \left(t-t_{p}\right)}{T}\right)\overset{N-1}{\underset{\underset{q\neq p}{q=0}}{\prod }}\frac{\sin\left(\frac{\pi \left(t-t_{q}\right)}{T}\right)}{\sin\left(\frac{\pi \left(t_{p}-t_{q}\right)}{T}\right)}$$

When N is an odd number,

(7) $$h_{p}\left(t\right)=\overset{N-1}{\underset{\underset{q\neq p}{q=0}}{\prod }}\frac{\sin\left(\frac{\pi \left(t-t_{q}\right)}{T}\right)}{\sin\left(\frac{\pi \left(t_{p}-t_{q}\right)}{T}\right)}$$

It can be observed that when the sampling points are odd and even, the reconstruction functions ${\left\{h_{p}\left(t\right)\right\}}_{p=0}^{N-1}$ are slightly different. Actually, the aim of this difference is to ensure the function to span a entire space of the periodic band-limited signal, so that the set of the function can constitute a basis for the space. This means that any x(t) can be expressed with this set of function, so as to achieve the purpose of reconstruction.

## 3. Proposed Method

Aiming at the nonuniformly distributed data obtained with low sampling rate from the servo control and drive system, this paper will discuss the proposed method in three parts: reorganization based on RES, reconstruction and error analysis.

### 3.1. Reorganization of Sampling Points Based on RES

For the purpose of increasing the sampling rate and rearranging the points within the same period, it is essential and important to obtain the precise period of the signal. We need to resample the initial samples to obtain a uniformly spaced sequence of samples firstly. Usually, the period of the signal is determined by the fundamental frequency in the frequency spectrum, but the error introduced by resampling will cause great interference in this step. Instead, we choose to find the peak frequency from the envelope spectrum to obtain the period of the signal. In this type of spectrum analysis, when the signal has poor spectrum accuracy due to errors such as the fence effect, the accurate frequency is often difficult to obtain. In this case, the frequency correction method [24,25] can be used to obtain the true value of the required correction frequency.

After getting the signal period, we next determine the position of the sampling point in the recombined signal. Let ${\left\{x\left(t_{i}\right)\right\}}_{i=1}^{N}$ be N nonuniform sample points to be reconstructed, and $t_{i}$ be the time corresponding to the *i*th point, $T_{0}$ is the period obtained in the previous step. The time of the new sequence after recombination is given by Equation (8), where mod is the remainder of $t_{i}/T_{0}$ and sort is to arrange these points from small to large.

(8) $$tr_{i}=\mathrm{sort}\left[t_{i}mod\left(T_{0}\right)\right]$$

### 3.2. Reconstruction of Nonuniform Sample Points

After recombining the signals within the same period, the equivalent sampling rate will increase to several times the original sampling rate. Next step, we introduce the reconstruction theory described above to complete the reconstruction of nonuniform sample points.

The stability of reconstruction [23] is represented by the condition number which can be denoted by

(9) κ=B/A

where B and A are the maximum and minimum eigenvalues of the aforementioned correlation matrix $R_{ij}$. κ is an index that is only related to the distribution of sampling points. The difference in the distribution of sampling points has a great influence on the stability of the reconstruction function. Specifically, when the distance between sampling points is very small or the gap is very large, the reconstruction function becomes unstable, and it is difficult to obtain an ideal reconstruction result.

In the step of recombining all the sampling points in one cycle, the positions of the sampling points can be distributed anywhere in the interval [0 *T*_0_]. According to the foregoing discussion, if there is a case where the distance between sampling points is close or far, the stability of the reconstruction algorithm will be greatly affected. An excessively large condition number κ may even cause reconstruction failure. The proposed solution is to perform a down sampling on the recombined sampling points. We select these points with the smallest deviation compared with the uniformly distributed sampling sequence and discard the remaining sample points with large deviations that do not contribute much to signal recovery. Finally, we use these samples that we selected as the input of the reconstruction algorithm. The selected sample points are brought into the reconstruction algorithm to realize signal reconstruction.

### 3.3. Reconstruction Error

There are several evaluation methods for signal reconstruction error, such as mean square error and mean absolute error. In this paper, mean absolute percentage error (MAPE) index is used to evaluate the accuracy of reconstruction.

The definition of MAPE is given by Equation (10), where $y_{r}$ is the amplitude of the reconstructed signal and y is the amplitude of the original signal. MAPE indicates the degree of deviation between the reconstructed signal and the actual signal. The smaller the deviation, the better the accuracy of the reconstruction algorithm

(10) $$\mathrm{MAPE}=\frac{1}{N}\overset{N}{\underset{i=1}{\sum }}\left|\frac{y_{r}\left(i\right)-y\left(i\right)}{y\left(i\right)}\right|*100\%$$

### 3.4. Flow Chart

**Figure 2:**
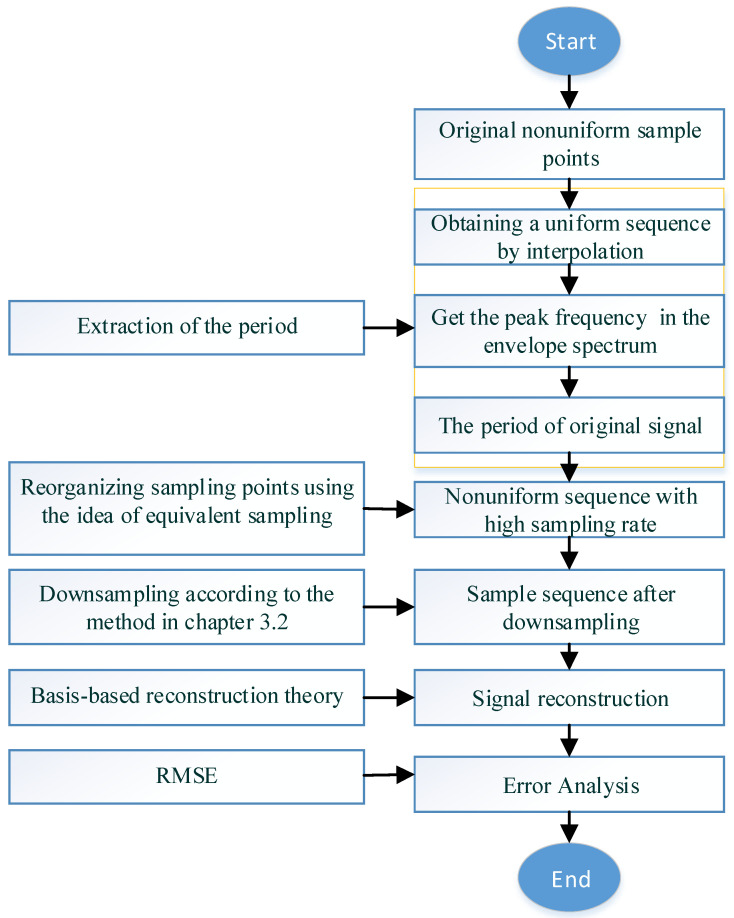
Flow chart of the proposed method.

As described above, the steps of the method proposed in this paper are as the follows and the flow chart of the method is shown in Figure 2.

1. Obtaining the original non-uniform, sub-Nyquist rate sample sequence to be reconstructed.
2. Uniformly resampling these nonuniform samples.
3. Calculating the period of the original waveform through the envelope spectrum of the resampled signal according to Section 3.1.
4. Regrouping sampling points from multiple periods into one period by using Equation (8).
5. According to the criteria in Section 3.2, some sampling points are selected as the input of the reconstruction algorithm through down sampling.
6. According to basis-based reconstruction theory, the discrete signal is reconstructed into a continuous signal.
7. Analyze the error of the reconstructed signal.

## 4. Simulation Verification

The simulation signals of periodic reciprocating motion of robot are constructed to verify the effectiveness of the above methods. During the reciprocating motion of the robot, the movement of the robot arm is divided into four stages.

The first stage is that the robot arm accelerates from the standstill to the maximum speed, and then slowly decelerates to a speed of 0, at which time the movement of robot arm reaches the end position.

The second stage simulates the state where the robot arm reaches the end position and stops for a period of time.

In the third stage, the robot arm starts to rotate from the end position, the speed increases to the maximum and then decreases to 0. The robot arm returns to the initial position.

The fourth stage simulates that the robot arm stops moving for a period of time after returning to the initial position, and then enters the first stage, cyclically reciprocating.

**Figure 3:**
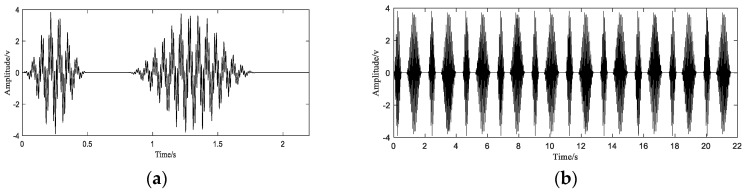
A full-cycle simulation signal (**a**) and simulation signal after extension (**b**).

In order to simulate the characteristics of this movement, the simulation vibration signal is now constructed as follows. Two sinusoidal signals with frequencies of 70 Hz and 15 Hz are used to simulate different frequency components in the signal. Due to the influence of the robot’s speed in the first and third stages of the robot arm’s movement, the robot arm should exhibit a relatively obvious amplitude modulation phenomenon on the waveform. A sine wave with a frequency of 2 Hz and 1 Hz is used as the modulation signal to simulate this phenomenon. Suppose the period of the robot arm movement is $T_{0}=2.2\;s$, and the time that the robot arm stays at the initial position and the end position is 0.4 s and 0.3 s, respectively. The simulation waveform of one movement cycle of the robot arm is shown in Figure 3a. Then the signal is periodically extended to obtain a signal of 10 cycles, the signal after the extension is as shown in Figure 3b.

**Figure 4:**
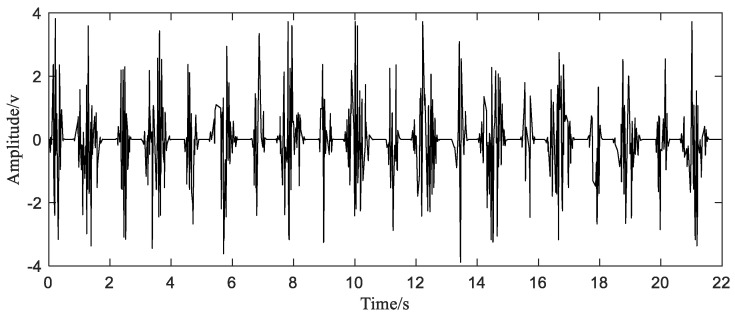
The initial signal with sub-Nyquist sampling rate.

In this simulation, the original sampling points are constructed by randomly generating 1200 points at arbitrarily intervals in the interval [0 $10T_{0}$]. The equivalent sampling frequency of these sample points is 54.5 Hz, which satisfies the condition of sampling at sub-Nyquist frequency. The initial signal with sub-Nyquist sampling rate is shown in Figure 4. Next, we use these nonuniform sampling points to reconstruct the original signal (Figure 3).

**Figure 5:**
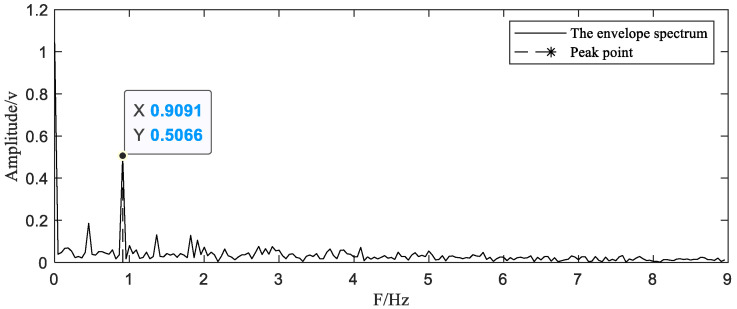
The envelope spectrum of the signal after resampling.

After uniformly resampling through interpolation, we calculate the period of the original signal from the peak frequency $F_{p}$ in the envelope spectrum of these pseudo sampling points. The envelope spectrum is shown in Figure 5. A period of the original signal contains two similar parts, so the time corresponding to the peak frequency is half of the period of the original signal. The signal period can be obtained according to Equation (11).

(11) $$T_{0}=2/F_{p}$$

It can be observed from Figure 5 that the peak frequency $F_{p}=0.9091\;\mathrm{Hz}$. $T_{0}=2.19998\;s$ can be calculated by Equation (11), which is very close to the true period of the original signal.

**Figure 6:**
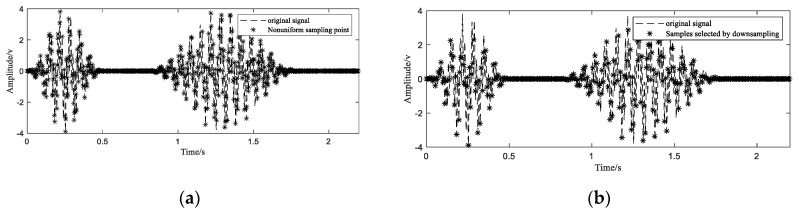
Reorganization of sampling points (**a**) and screening of sampling points (**b**).

After the period of the signal $T_{0}$ is determined, according to the corresponding positions of the sampling points in their respective periods, they are transferred to the first period. The new time position tr is determined by Equation (8). In order to avoid the failure of reconstruction caused by the distance between the sampling points, the sample point with the smallest deviation from a uniformly distributed sequence are selected from all the regrouped samples. The highest frequency of the simulation signal is 70 Hz. Theoretically, as long as the average sampling rate is greater than or equal to 140 Hz, the original signal can be recovered. After recombining 1200 points of 10 cycles into one cycle (Figure 6a), there are a lot of redundant sampling points. As shown in Figure 6b, 340 nonuniform sample points closest to the uniform time series are selected out from 1200 samples to ensure the distribution of nonuniform samples and to discard those points with very close or far spacing.

**Figure 7:**
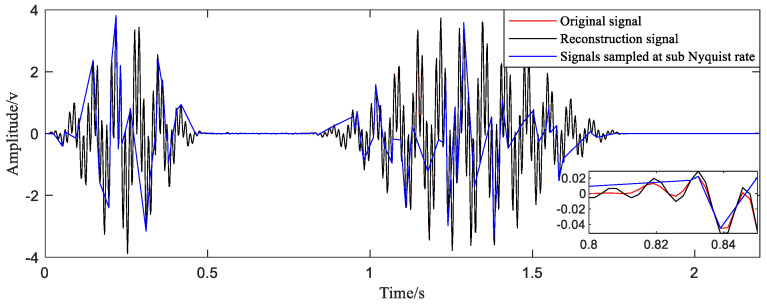
Comparison between reconstructed signal and original signal.

Finally, these points are taken into the reconstruction function when the sampling points are even (Equation (6)), and the signal of one period after reconstruction is obtained. As shown in Figure 7, compared with the original signal, only a few points of the reconstructed signal deviate from the true value, and the deviation is small. The value of MAPE calculated according to Equation (10) is 0.27%. Such a small value indicates that the accuracy of the reconstructed signal is quite high.

## 5. Reconstruction of Actual Signal

**Figure 8:**
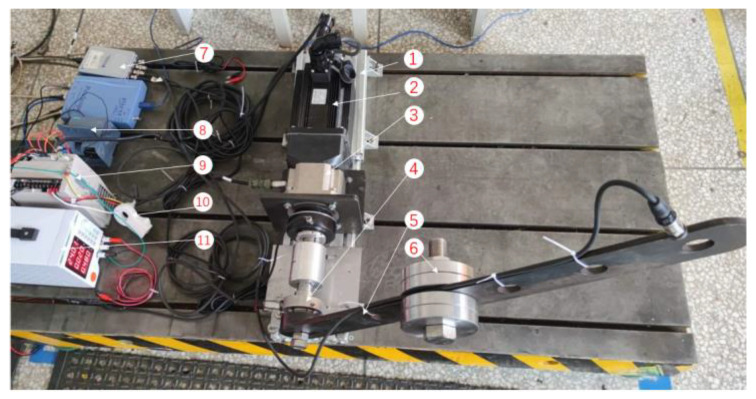
Swing arm test stand.

The current signal collected from the arm swing test stand is used to verify the effectiveness of the proposed method. As shown in Figure 8, the structural part of the stand is composed of base, servo motor, reducer, bearing seat, swing arm and other components. For the control part, Siemens PLC is applied to implement the periodic reciprocating movement of the robot arm swing. The size of the weights and its installation position on the swing arm can be adjusted to simulate the change of load. The function of the test stand is to collect the current and vibration signals of the swing arm in the whole life test process to study the decline characteristics of the reducer. The current signal is collected by the NI9234 acquisition card with a sampling rate of 256 Hz and the period of swing movement is 5 s. The names of all components of the test stand are given in Table 1.

**Figure 9:**
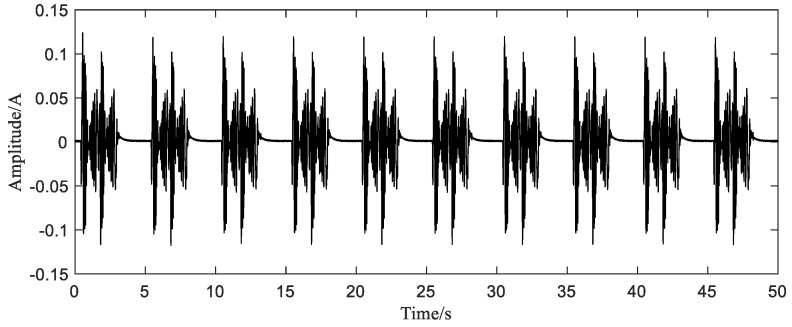
Ten cycle current signal.

**Figure 10:**
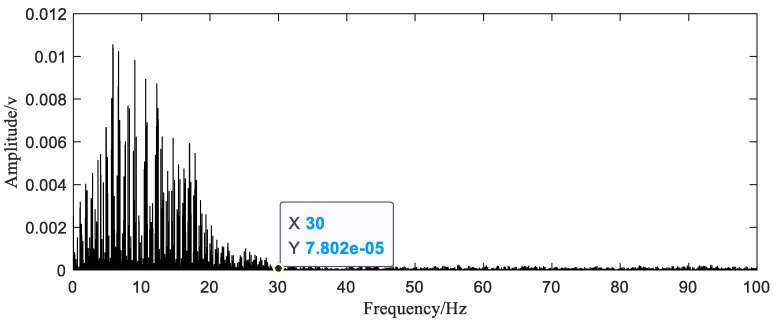
Spectrum of original current signal.

As in simulation, we take 10 full-cycle current signals (Figure 9), and then obtain the initial nonuniform sample sequence for reconstruction by down sampling the original data. The number of sampling points is 1000. By observing the spectrum of the original signal, it can be seen that the highest frequency of the signal is around 30 Hz, as shown in Figure 10. Therefore, the average sampling rate 20 Hz still satisfies the sub-Nyquist sampling rate.

**Figure 11:**
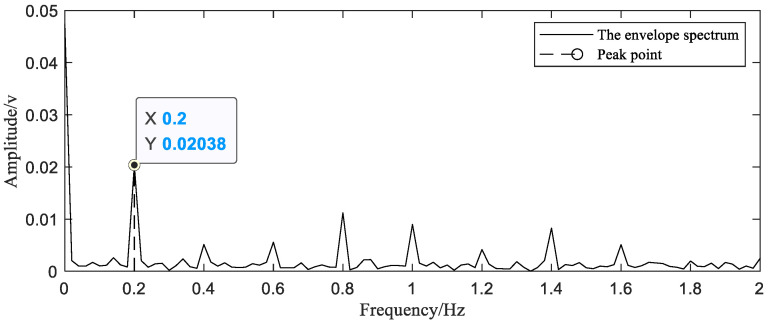
The envelope spectrum of the current signal after resampling.

After uniform resampling, the peak frequency of 0.2 Hz can be seen in the envelope spectrum (Figure 11). The current signal does not show two very similar waveform components in one cycle as in the simulation. Therefore, the signal period 5 s is obtained by Equation (12),

(12) $$T_{0}=1/F_{p}$$

**Figure 12:**
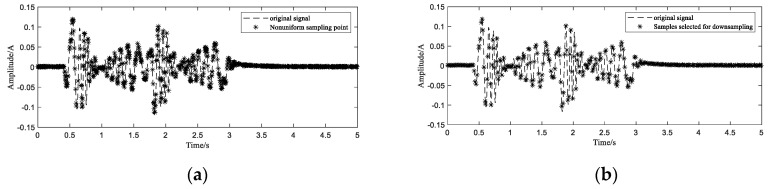
Reorganization of sampling points (**a**) and screening of sampling points (**b**).

Referring to RES, after recombining 1000 samples in the same cycle (Figure 12a), in order to improve the stability of the reconstruction algorithm and avoid reconstruction errors, the samples are down sampled. As described in the previous simulation section, samples with small interval changes shall be selected as much as possible. Theoretically, if a signal with a sampling rate greater than 60 Hz can recover a signal with a maximum frequency of 30 Hz, then we need at least 300 sampling points within a period of 5 s. In order to improve the accuracy of reconstruction, 320 samples are selected from 1000 points, as shown in Figure 12b.

**Figure 13:**
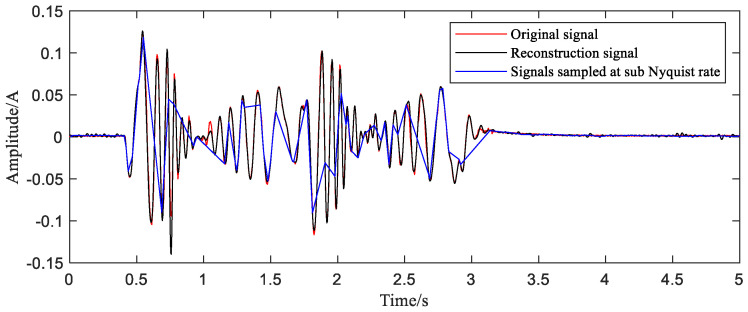
Comparison between reconstructed signal and original current signal.

**Figure 14:**
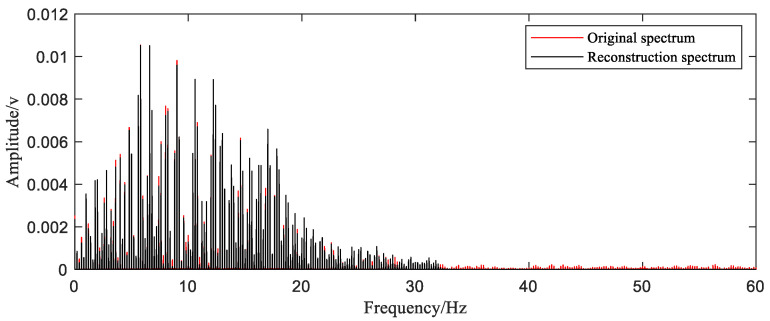
Spectrum comparison.

The last step is to bring samples selected into the reconstruction function and obtain the reconstructed time-domain waveform. In order to show the quality of the reconstruction effect more intuitively, the time-domain comparison chart (Figure 13) and frequency-domain comparison chart (Figure 14) are given.

The time-domain waveform of the reconstructed signal is basically consistent with the original signal except for a small number of points that are slightly offset. We can observe in the frequency spectrum that the reconstructed signal basically covers all the components of the original signal with slight differences of amplitude. The frequency components higher than 30 Hz in the original signal spectrum are not reflected in the reconstructed signal, but this part of the frequency component has very low energy and can be directly ignored. The MAPE value calculated by Equation (9) is 0.66%. The results of the reconstruction are generally satisfactory.

## 6. Conclusions

In order to solve the difficulty of signal reconstruction due to the low sampling rate and uneven sampling of the data obtained from the servo system, this paper proposes a method to recombine the sample points in the same period by calculating the period of the signal, and then using the basis-based reconstruction theory to achieve signal reconstruction. According to the characteristics of the reconstruction algorithm, the method of down sampling with slightly redundant sample points improves the stability of reconstruction. In this paper, the simulation signal of the reciprocating motion of the robot arm and the actual collected current signal are used for down sampling to obtain the initial non-uniform samples. Both the simulation and experiment parts show the comparison results of the time-domain waveform and frequency spectrum of the reconstructed signal and the actual signal. We can see that the time domain of the reconstructed signal deviates very little from the actual signal (MAPE values are all less than 1%), and there is no loss of main frequency components in the frequency spectrum. These results prove the effectiveness of the method.

## Figures and Tables

**Table 1:** Description of the parts of the test stand.

Number	Name of Parts	Number	Name of Parts
1	base	2	AC PMSM
3	planetary gear reducer	4	bearing seat
5	swing arm	6	weights
7	acquisition card	8	Siemens PLC
9	servo driver	10	current sensor
11	power		
